# Pathways into and out of homelessness among people with severe mental illness in rural Ethiopia: a qualitative study

**DOI:** 10.1186/s12889-021-10629-8

**Published:** 2021-03-22

**Authors:** Caroline Smartt, Kaleab Ketema, Souci Frissa, Bethlehem Tekola, Rahel Birhane, Tigist Eshetu, Medhin Selamu, Martin Prince, Abebaw Fekadu, Charlotte Hanlon

**Affiliations:** 1 King’s College London, Institute of Psychiatry, Psychology and Neuroscience, Health Service and Population Research Department, Centre for Global Mental Health, London, UK; 2 King’s College London, King’s Global Health Institute, London, UK; 3 Independent Researcher, Ethiopia; 4 Addis Ababa University, College of Health Sciences, School of Medicine, Department of Psychiatry, WHO Collaborating Centre for Mental Health Research and Capacity-Building, Addis Ababa, Ethiopia; 5 Addis Ababa University, Centre for Innovative Drug Development and Therapeutic Trials for Africa (CDT-Africa), College of Health Sciences, Addis Ababa, Ethiopia; 6 Department of Global Health & Infection, Brighton and Sussex Medical School, Brighton, UK

**Keywords:** Homelessness, Severe mental illness, Low- and middle-income countries, Global mental health, Psychosis, Substance use

## Abstract

**Background:**

Little is known about the pathways followed into and out of homelessness among people with experience of severe mental illness (SMI) living in rural, low-income country settings. Understanding these pathways is essential for the development of effective interventions to address homelessness and promote recovery. The aim of this study was to explore pathways into and out of homelessness in people with SMI in rural Ethiopia.

**Methods:**

In-depth interviews were conducted with 15 people with SMI who had experienced homelessness and 11 caregivers. Study participants were identified through their participation in the PRIME project, which implemented a multi-component district level plan to improve access to mental health care in primary care in Sodo district, Ethiopia. People enrolled in PRIME who were diagnosed with SMI (schizophrenia, schizoaffective disorder or bipolar disorder) and who had reported experiencing homelessness at recruitment formed the sampling frame for this qualitative study. We used OpenCode 4.0 and Microsoft Excel for data management. Thematic analysis was conducted using an inductive approach.

**Results:**

Study participants reported different patterns of homelessness, with some having experienced chronic and others an intermittent course. Periods of homelessness occurred when family resources were overwhelmed or not meeting the needs of the person with SMI. The most important pathways into homelessness were reported to result from family conflict and the worsening of mental ill health, interplaying with substance use in many cases. Participants also mentioned escape and/or wanting a change in environment, financial problems, and discrimination from the community as contributing to them leaving the home. Pathways out of homelessness included contact with (mental and physical) health care as a catalyst to the mobilization of other supports, family and community intervention, and self-initiated return.

**Conclusions:**

Homelessness in people with SMI in this rural setting reflected complex health and social needs that were not matched by adequate care and support. Our study findings indicate that interventions to prevent and tackle homelessness in this and similar settings ought to focus on increasing family support, and ensuring access to acceptable and suitable housing, mental health care and social support.

**Supplementary Information:**

The online version contains supplementary material available at 10.1186/s12889-021-10629-8.

## Background

People with severe mental illness who experience homelessness are an especially vulnerable group worldwide. The co-occurrence of severe mental illness (SMI, schizophrenia, schizoaffective disorder and bipolar disorder [1]) and homelessness is higher than expected from the frequency of each condition in the general population and is associated with high levels of unmet need [2, 3]. Recent global reviews and meta-analyses found significantly higher pooled prevalence of psychotic disorders among people experiencing homelessness in studies from low- and middle-income countries (LMICs) than high-income countries (HICs) (29% vs. 19%) [4, 5], with high levels of suicidality [6] and a heavy burden of comorbid physical health and substance abuse problems [7].

This problem is very serious worldwide, and is further complicated in LMICs where health and social services make little or no provision for people with SMI who are homeless, placing them at heightened risk for neglect, suffering and abuse [3]. Differing family support networks and other cultural factors likely impact on the composition of homeless populations in LMICs, however there has been little very literature exploring this to date and few descriptions of formal services for people who are homeless [3].

What is known about pathways into and out of homelessness among people with SMI is based on studies almost exclusively conducted in high-income settings [8]. In these studies, pathways into homelessness are complex and often result from the interaction of multiple factors operating at different levels: individual, family, community, and society [9, 10]. Individual-level factors identified as influencing pathways into and out of homelessness include the symptoms of mental and/or co-morbid physical ill health and substance abuse [11, 12] and their associated impacts on the person’s ability to function in their daily life [13–16]. Abrupt withdrawal of family and community support, and strain arising from these relationships (stigma, violence and abuse and other conflicts) have been identified as precipitants [10]; however, families and communities can help people with SMI to exit homelessness when they provide housing (transitional and/or long-term) and assistance in meeting other basic needs and offer emotional and financial support [17]. Structural barriers to family and community support include intergenerational poverty and poorly funded social services, which necessitates government action on the societal level. Other societal-level factors include the availability of housing stock and the accessibility of informal and formal sectors of help, including health and social care and supported housing [18]. Finally, socioeconomic status and financial reserves [19], and access to informal and formal sectors also play a key role [10, 20].

In social science literature from HICs, the word “pathways” has been used to describe the mechanisms for people to become homeless, primarily emphasizing structural causes for homelessness [10]. This term was reinforced in the public health literature in the early 2000s as the “Pathways to Housing” model gained traction worldwide as the most successful evidence-based intervention for helping people who are homeless and have SMI and/or co-occurring substance abuse problems return to permanent stable housing [21, 22]. In this paper, we use the word “pathways” into and out of homelessness to describe the range and variety of experiences of our study participants in terms of their entering and exiting homelessness.

Homelessness in relation to mental illness in LMICs has received little research attention to date. In a recent review [3], only two qualitative studies from LMICs had examined the phenomenon of homelessness in people with SMI [23, 24]. Neither of these studies was focused on pathways into and out of homelessness, neither was conducted in sub-Saharan Africa, both were restricted to urban settings, and only one included interviews with those with lived experience of homelessness and SMI [24]. We conducted a qualitative study with people with SMI who had experienced homelessness in a rural Ethiopian setting with the objective of identifying and examining pathways into and out of homelessness in order to inform future interventions.

## Methods

### Study design

A qualitative study comprising in-depth interviews with people with SMI who had experienced homelessness and their caregivers.

### Setting

The study was conducted in Sodo district, in the Gurage administrative zone of the Southern Nations, Nationalities and Peoples’ Region of Ethiopia (SNNPR). Sodo is approximately 100 km from Ethiopia’s capital city, Addis Ababa, and has a population of approximately 162,000 inhabitants [25]. Most of the population live rurally and are engaged in subsistence agriculture (88%) [26]; approximately 45% of the population are estimated to be literate [27]. Typically, households are composed of a mix of immediate and extended family members living alongside one another and sharing livelihood responsibilities [28].

Health services in Sodo at the time of the study included one newly operational primary hospital [29], eight health centres serving catchment areas of approximately 25,000 (rural) to 40,000 (urban) people and 58 health posts serving 3000–5000 people. These health care facilities are staffed mainly by nurses and health officers in the health centres and by health extension workers in health posts. A resource mapping exercise estimated there to be around 150 traditional healers operating in the district, including herbalists, witch doctors/sorcerers (‘*tanquaye’)*, traditional birth attendants, and traditional bonesetters [30].

Ethiopian Orthodox Christianity is the dominant religion in the district [29]. Biomedical care, traditional healers and holy water sites linked to the Orthodox Christian church are all relied upon for care for people with mental health problems. Holy water is accessed at specific sites for its curative properties, where people may reside for short or long-term stays; there are 27 of these sites located within the district [30].

At the time of the study, mental health care delivered at the primary care level had recently been introduced into the district as part of the Programme for Improving Mental Healthcare (PRIME) project [25]. Prior to the initiation of PRIME, people with mental health problems had to travel to a psychiatric nurse-run outpatient clinic in Butajira town (with approximate travel time of 30 min - 3 or more hours), or to Addis Ababa for inpatient psychiatric treatment or interventions for substance use disorders [31]. PRIME was a five-country research consortium that aimed to generate evidence on the integration and scale of up of mental health care within primary health care for priority conditions including SMI [32]. In PRIME, participatory methods were used to develop an integrated district level mental health care plan which was implemented in the community, in health facilities and at the level of the health system [25].

Community key informants were trained to identify people with potential severe mental illness and refer them to primary care settings where heath workers had been trained in the World Health Organization’s Mental Health Gap Action Programme [26]. As reported previously [33], a total of 1035 people with probable SMI or epilepsy were referred to primary care; *n* = 972 took up the referral and were assessed; 300 received a confirmatory diagnosis of SMI from a psychiatric nurse and entered the cohort study.

### Sampling

We defined homelessness as any experience of spending the night outside the home unsheltered overnight. This was intended to include a range of experiences and has been used previously in LMICs, including in rural China [34] and urban Ethiopia [35]. At the cohort baseline assessment, carried out at the time of initiating mental health care in primary health care services, participants with SMI were asked the following question, based on the Butajira Treatment Gap Questionnaire [36], as part of a lay interviewer administered questionnaire following clinical assessment and diagnosis with SMI: “Since the illness began, has there ever been a time when you stayed outside the house overnight in the fields, streets, or forest?”; participants who answered this question as “yes” were categorized as having experienced homelessness (*n* = 109, 36.3%) and formed the sampling frame for this study.

PRIME participants who had indicated that they had spent nights sleeping outside the home on the baseline questionnaire were identified using the electronic file linking ID number to participant’s names and were approached by PRIME research team members (data collectors and supervisors from the local area) who had already interacted with the person previously.

Our sampling of participants with SMI and history of homelessness was purposive and based on the following criteria: duration and types of homelessness experienced, gender, and location (rural (inaccessible to main roads) vs. peri-urban (proximate to the main road to Addis Ababa)).

We interviewed the registered caregivers of people with SMI where possible. In some cases, the person with SMI was too ill to be interviewed and we were only able to interview their caregivers; in other cases, caregivers were not available. We did not purposively sample caregivers for any characteristics. We interviewed caregivers with the informed consent of the person with SMI, first asking if there was anything they did not want us to discuss with their caregivers.

### Data collection

In-depth interviews (IDIs) were conducted between October 2017 – November 2018 by an Ethiopian research assistant with a master’s level degree and experience in public health fieldwork (KK) along with the first author, an American researcher with contextual knowledge of the study site and Amharic language ability. KK received training from the PRIME Ethiopia research coordinator (MS), who conducted the first two interviews with KK and CS in attendance.

Potential participants were told about the study aims and objectives both orally and in writing and, after agreeing to participate, provided written informed consent or a witnessed thumbprint from those who were illiterate. Not all study participants with SMI were in full remission, but all agreed to participate after being read the consent form and were judged by the interviewer to have the capacity to consent. We stopped interviews if participants were unable or unwilling to participate further.

The interviews were conducted at a convenient location for the respondent, including their own homes, the PRIME project office, a health facility or another community setting where privacy could be assured. Interviews lasted between 30 and 60 min. All participants received a payment of 100 birr as a reimbursement for their time (approximately $3–4).

The IDIs were structured around a topic guide which asked about people’s experiences moving into, through and out of homelessness as well as their ideas for the prevention of future homelessness and how to help other people with SMI who experience homelessness. Field notes were documented by CS working closely with KK. The audio recordings were transcribed into Amharic and then translated into English by KK, with footnoting to explain where text was ambiguous or included cultural references. Translations and footnotes were checked by another native Amharic speaker (SF) who has extensive experience of conducting fieldwork in rural Ethiopia. The interviews were stopped once theoretical saturation was reached in order to minimise the research burden on this vulnerable group.

### Reflexivity

As a white American female, the presence of the lead investigator (CS) at the interviews could have affected the responses of participants. Furthermore, KK also has outsider status from the community studied in terms of being identifiably from the capital Addis Ababa and having a high level of education. We attempted to acknowledge this and reflect on our roles by observing the dynamics established over the course of the interviews and adjusting accordingly.

### Data analysis

We conducted the study in an iterative manner, with preliminary analyses informing development of the topic guides.

Thematic analysis of the English translation of the IDIs was conducted using Opencode 4.0 for data management [37]. We used an inductive (data driven) approach to identifying themes. CS, RB and TE independently coded four interviews and a meeting was held to combine our inputs and draft a coding scheme. We did not check reliability of coding statistically. However, we did compare line-by-line coding of cross-coded transcripts and had detailed discussion about emerging codebooks to understand how different coders interpreted the data. Mostly this allowed for consensus adjustment of the codebook, but senior authors (CH and SF) were involved if there was uncertainty.

CS then coded the remaining interviews, refined the codebook further, discussed the final codebook with RB, TE, KK, and CH, and recoded all the interviews. CH provided oversight and supervision based on her expertise in qualitative methodology and experience working in the study site.

CS collated the codes into potential themes and sub-themes, identifying repeated patterns of meaning throughout the dataset as related to the research questions, created a map of how themes were related to one another and discussed and finalised this with CH, KK, and SF. CS extracted the relevant sections of each interview into an excel spreadsheet according to themes and sub-themes and finally reread the full transcripts to check that the final thematic framework reflected the totality of the data contained in the interviews. A comparative analysis of themes was carried out with respect to key participant characteristics (person with SMI vs. caregiver, gender, and typology of homelessness).

### Ethical considerations

Ethical approval was obtained from both the Addis Ababa University College of Health Sciences Institutional Review Board (084/11/Psy) and from King’s College London (HR-1617-3691). If the interviewer had any concerns about the mental health status of the person, the participant was encouraged to attend services, with involvement of caregivers as needed and only with consent.

## Results

We interviewed 15 people with lived experience of SMI and homelessness (*n* = 12 men; *n* = 3 women) and 11 caregivers (*n* = 6 men; *n* = 5 women). See Tables 1 and 2 for characteristics of the study sample. There were more men with SMI interviewed than women: this was because more men had experienced homelessness and because the women who had been homeless were more inaccessible and living in more remote areas. One potential participant refused to participate, one was chained at home and four others were too ill to be interviewed. All participants were diagnosed with schizophrenia or another primary psychotic disorder. See supplementary file 1 for information about the living situation of study participants at the time of interview.

**Table 1:** Socio-demographic characteristics of people with lived experience of SMI and homelessness

People with lived experience of SMI and homelessness							
Interview	Gender	Age	Marital status	Years of education	Residence	Type of homelessness experienced	Linked interviews
IDI 3	Female	40	Divorced	0	Urban	Long-term	IDIs 1, 2
IDI 7	Male	38	Married	6	Urban	Long-term	IDI 6
IDI 11	Male	56	Widowed	9	Urban	Long-term and intermittent linked with alcoholic relapse	IDI 9
IDI 12	Male	44	Single	0	Rural	Intermittent	
IDI 13	Male	28	Single	9	Urban	Intermittent	
IDI 14	Male	35	Married	8	Urban	Single homeless episode	IDI 15
IDI 16	Male	40	Single	6	Rural	Intermittent	
IDI 18	Male	30	Single	0	Rural	Intermittent	
IDI 19	Male	45	Married	6	Rural	Intermittent	
IDI 20	Male	50	Married	7	Rural	Intermittent homelessness reported by caregiver; person with SMI reported that they had stayed in other people’s homes	IDI 21
IDI 22	Male	28	Single	5	Rural	Intermittent	
IDI 23	Male	38	Single	5	Rural	Long-term	IDI 24
IDI 24	Male	25	Single	4	Rural	Intermittent	IDI 23
IDI 25	Female	28	Married	7	Urban	Intermittent	
IDI 26	Female	30	Single	9	Rural	Intermittent

**Table 2:** Characteristics of caregivers for people with lived experience of SMI and homelessness

Caregivers			
Interview	Gender	Relationship to person with SMI	Linked interviews
IDI 1	Female	Community member/friend	IDIs 2, 3
IDI 2	Female	Community member^a^	IDIs 1, 3
IDI 4	Male	Community member^a^	
IDI 5	Female	Sister^a^	
IDI 6	Male	Community member^a^	IDI 7
IDI 8	Male	Employer	
IDI 9	Male	Nephew^a^	IDI 11
IDI 10	Male	Father^a^	
IDI 15	Male	Father^a^	IDI 14
IDI 17	Female	Mother^a^	
IDI 21	Female	Wife^a^	IDI 20

^a^official PRIME caregiver

The sample comprised people with extended episodes of homelessness (long-term homelessness, at least a year in duration) as well as those who experienced briefer periods of episodic (intermittent) homelessness. Long-term homelessness ranged from 1 year to over 20 years. Intermittent homelessness was characterized by people cycling between living on the streets and their original homes. Some of those experiencing intermittent homelessness remained in areas near to their homes of origin, others traveled far from home, and/or to multiple different locations during these episodes.

### Pathways into homelessness

Experience of mental ill health and family conflict and relationship strain were prominent in the narratives describing pathways into homelessness. Escape from coercion and the desire for a change in one’s environment also emerged as an important theme leading to homelessness in many cases.

#### Experience of mental ill health

Both people with SMI and their caregivers described the onset or escalation of mental health problems prior to homelessness. The nature of these problems was grouped into the following categories: confusion; feelings of anxiety or restlessness; strange/disturbing experiences; substance use; and suicidality.

Confusion was articulated as ‘not knowing’, being aware or remembering, being confused, or doing things for ‘inexplicable reasons’ or ‘haphazardly’:

“My mind went blank even when I was in the middle of a conversation. Then the illness became worse and I started to lose control of myself like a dead person. I became very ill.” (IDI 7)

Worry, stress, and restlessness were commonly described using the term ‘*chinket*’, conveying a feeling of emotional distress. Sometimes this occurred in the context of unusual experiences:

“I felt anxious and I couldn’t settle down in the house… Something I didn't know talked to me…I left the house because I thought that [the anxiety] will leave me when I leave the neighborhood.” (IDI 23)

The onset of mental illness and homelessness was also explained by people with SMI in terms of bewitchment.

“I had a disagreement with someone, and they did *‘asmat*’ [a spell] on me. They buried a deer's neck in my farm. They cut the neck and buried it in my workplace. I became ill and left.” (IDI 7)

Alcohol and other substance use were commonly linked with spending nights outside of the house. One caregiver described a pattern of his uncle drinking alcohol, stopping medication, relapsing and sleeping on the streets:

“The biggest problem we are experiencing now is that his illness relapses and he becomes very ill when he… drinks...His life becomes terrible, because he refuses to take the medication, leaves the house and starts living out in the streets...” (IDI 9)

#### Family conflict and relationship strain

Family conflict prior to homelessness was recounted by people with SMI and caregivers and was linked with the experience of mental ill health and/or behavioural changes. In some cases, people with SMI spoke of family conflict leading to mental health problems and their perceived need to leave the home; in other cases, changes in the behaviour of the person with SMI were reported to be triggers for conflict. Family conflict was expressed in marital, nuclear and extended family relationships.

Some people with SMI described feeling alienated, excluded, and/or exploited in the family home:

“It was nothing but toiling. Only toiling…They were nagging me. They didn't want me to live in the house.” (IDI 3)

Conflict within marriages was commonly reported. One woman with SMI described her husband’s substance abuse and irresponsible behaviour as driving her into homelessness.

Other aspects of family interactions described by participants as triggers for homelessness were abandonment, neglect and violence. One man with SMI described being abandoned abruptly by his uncle with whom he was living, leading to a homeless episode. Violence in the home and extended family was reported, with people with SMI as occasionally perpetrators, but more described as being victims of violence. In only one case, a woman with SMI described acting violently. She subsequently left her home due to guilt, feeling that she deserved to be punished:

“I said to myself, ‘How could one beat one’s own mother?’ and left the house... I felt bad that I beat my mother and I decided that I should be eaten alive by hyenas.” (IDI 26)

For some, unmanageability of the person with SMI was reported to have overwhelmed the family unit. It was common that families had exhausted their financial and emotional resources, as well as their treatment options (both holy water and within formal health care) prior to the person becoming homeless. Conflicts over adherence to medication were reported, as were conflicts over drinking alcohol.

Several people with SMI described financial issues as important pathways into homelessness via impact on family relationships and a sense of having lost one’s worth and value; financial problems included job loss, loss of money, and generally feeling like a financial burden to their families.

“They are already poor…My family spent a lot of money...They lost their property to seek treatments for me. So I decided that they shouldn't know when I leave the house and I left the house.” (IDI 21)

#### Escape/change in environment

Respondents described the “escape” of people with SMI from formal health care, holy water treatment, restraint and/or family monitoring. Caregivers were more likely to describe people with SMI as “escaping” or “slipping our watch”, while those with SMI were more likely to describe mental ill health as leading them to leave home and/or treatment. One caregiver described how her brother escaped from a psychiatric facility in Addis Ababa; several others described escape from holy water treatment. Escape led to situations of high risk and/or vulnerability, including imprisonment.

Escape was used to justify the use of restraint. One man reported how:

“I slept in the forest at night. The holy water attendants feared that I would be eaten alive by hyenas and they bought a chain and a padlock, they chained me and kept me behind a closed door.” (IDI 22)

Restraint was also used in the transport of people with SMI to receive treatment and several people tried to escape during this process.

Other people with SMI left their homes because they associated their mental health problems with their physical environments, and in other cases, they described a preference for homelessness:

“It is my way of life…I am a street dweller. That is just my way of life. I rove the street and live outside the house.... I can't live in the house.” (IDI 24)

Discrimination from the community in terms of housing and general social ostracization contributed to homelessness for several people. Several were evicted from rented houses when landlords discovered their mental illness. One found the gossip in the community about his condition to be unbearable:

“People gossiped about my illness…After that, I decided that it would be better for me to live in a place where no one knows about my illness...” (IDI 22)

#### Pathways out of homelessness

The most salient pathways out of homelessness were medical treatment as a catalyst to accessing other supports, family and community intervention and support, and self-return.

#### Medical treatment

Receiving medical treatment for both mental and physical illness was a contributing factor to ending homelessness when it also acted as a starting point for people to access other supports. Health care services were accessed both within and outside the district. Psychiatric treatment included hospitalization in Addis Ababa and outpatient treatment provided more locally with injectable and oral medication; at least one homeless man reported being given treatment involuntarily.

One non-related caregiver described the return to housing of a woman with long-term homelessness who required hospitalization for typhoid and was given mental health care during her hospital stay:

“I brought her here for another illness… It was very difficult to give her treatment, but she was not aggressive because she was very ill…My neighbours who knew that I am worrying told me that her son had built a house for her. I didn't even worry about getting his permission and went to the compound…I broke into one of the houses and cleaned it…She entered the house that day...” (IDI 2)

Another person with SMI with long-term homelessness was given involuntary treatment. This man had been assaulting women in the town and there were concerns from the community. His behaviour improved with the treatment. After witnessing his improvement, the community mobilized resources to provide him with sustainable housing:

“He had to be held by eighteen people to be injected with the medication…He showed a significant improvement and there were only eight of us needed when he was injected for the second time…Only three people held him for the third time…Surprisingly, he remembered the exact date and went to the hospital [by himself, to get the next injection]. When I saw that, I started worrying [about finding a house for him].” (IDI 4)

Others with SMI sought out treatment for themselves, and said that this was the reason they were able to return to their homes:

“I heard that some health professionals have come…I went there and got medical treatment…Now, I go every month by myself and bring the medication…I got back home after I started medical treatment in the hospital.” (IDI 13)

#### Family and community support

Family and community support were usually essential in either encouraging or forcibly returning people to housing. Community members helped people who were homeless in different ways, for example, in the case of the woman who was given treatment during her hospitalization. Several members of the community supported her while she was homeless and continued to help her transition back to housing. They took ongoing measures until she became more accustomed to her new living situation, including helping her with basic needs and demolishing the temporary shelter where she had previously been living on the streets to disincentivize her from returning to homelessness.

Family members were usually the central figures in helping people to return home. One man with SMI was brought home to his family after he escaped holy water treatment and traveled to Addis Ababa where he was living on the streets:

“I paid for radio announcement. I finished all my money to find him. Luckily, people found him using the signs I gave them and they brought him back home. I tie him up and keep him in the house after that.” (IDI 10)

People with frequent homeless episodes described a pattern of being taken home habitually by both family and community members. Sometimes searching would occur on a daily basis, or for prolonged periods of time in the case of people with long-term homelessness. Searching and retrieving people in the evenings after nightfall was reported; caregivers described being fearful of the risks to the person of sleeping outside as well as to themselves:

“Something bad could happen to me when I go out to see him at night. But, I do that because I am his mother. I did this for eight years...I went to the place where he chews khat and brought him back home.” (IDI 17)

Occasionally, strangers allowed people who were homeless to stay with them. In one extraordinary case, a man traveled to a faraway area in search of Orthodox Christian holy water and stayed with a Muslim family living nearby. They hosted him and facilitated his return home when he was ready:

“They said they are Muslims and don't go to the holy water place, but they showed me where it is found. They told me that I could bring the holy water home and pour it on my body. I lived with them for one month…Then, I told them that I left my house because of my illness and that my parents would be mourning my death if they couldn't find me. So they gave me money for transportation and I came back home.” (IDI 22)

In other cases, temporary shelter and prepared food was provided for homeless study participants within Orthodox church compounds (outside of the context of the holy water treatment previously described), wherein people who were homeless would spend nights in the compounds, leave in the daytime and return at nightfall. The most common way for non-related people to help people who were homeless was through the provision of food and water.

#### Self-return

Self-return was a common pathway in returning to housing among people who experienced intermittent homelessness. One caregiver described her sister’s patterns of staying outside the home when she is unwell, and how the family responded:

“My mother doesn't lock the door when she sleeps because she knows that she usually will come back home…But she says that she stays outside late because she wants to be eaten alive by hyenas...Actually we search for her when she stays outside late…If we don't find her, we just wait for her until she comes back home by herself. She usually comes back home after one night.” (IDI 5)

People with SMI described exercising personal agency in making decisions about when to both leave and return home:

“I was fine when I lived outside the house. So I thought that I would be fine if I came back home. That was why I came here…I returned home by myself.” (IDI 23)

Another man described this in poetic terms:

“I just wandered the street and the road led me straight to my house…Yes. I came here by myself. The road led me straight to my house.” (IDI 24)

Self-return also emerged as a theme in connection with intermittent homelessness and alcohol abuse and occasionally led to serious consequences, such as the loss of livelihood:

“Yes, he went there sometimes, to drink…when he felt anxious…He got back home by himself. He put firewood on the donkeys’ back and went there. But, he left the donkeys there and came back home alone” (IDI 21)

## Discussion

To the best of our knowledge, this is the first qualitative study focused on the pathways traveled into and out of homelessness among people with SMI in a rural, low-income country setting. Our findings help to characterise important pathways and inform the development of prevention and intervention strategies for this vulnerable group. Family situations and relationships were crucial for both becoming homeless and exiting from homelessness, interfacing with access to mental health care, poverty and community support.

### Pathways into homelessness

People with SMI and their caregivers placed contrasting emphases on the exact mechanisms which precipitated homeless episodes. While people with SMI were more likely to describe their experiences of mental ill health, caregivers focused more on how their coping resources had been exhausted. Conflicts within families were reported by all study participants.

In this setting, the major portion of the burden of care generally falls to families [38]; access to mental health care depends on families [39, 40], and there is almost no formal social support. Supporting the whole family unit has been recommended as the best approach to ensure the best care and treatment for people with SMI in this context [41]. This entails not only consistent financial support, for example from community-based health insurance schemes [42], but also psychoeducation for family members about the nature of the illness and how to reduce stigma [38].

Studies from HICs and India have indicated that “expressed emotion” in the family, namely criticism, hostility and emotional overinvolvement/intrusiveness [43], contributes to the exacerbation of mental health problems in those with SMI [44]. More needs to be understood about the precise communication patterns that are culturally salient in this Ethiopian setting. Family interventions, including a focus on expressed emotions, are recommended as an evidence-based practice in HICs [45] and have been adapted for several LMIC contexts and shown to improve certain mental health and social outcomes [46]. Family mediation drawing on local practices may help to resolve some of the underlying issues among family members.

Experiencing mental ill health was reported by all study participants with lived experience of homelessness. Evidence from previous studies in the area indicates that access to medication can reduce caregiver stress and burden, which could in turn help to moderate some of the conflicts which trigger people to leave their homes [47]. However structural barriers, in particular poverty contributing to non-affordability of ongoing care, prevented people with SMI from easily engaging in ongoing care [40]; this is likely to be pronounced among those who have been or are currently homeless. It is not possible to know how rural residence impacted on homelessness from this qualitative study, but future studies should examine any relationship between proximity to services and risk of homelessness in order to quantify risk and raise awareness of the need for locally-provided care.

Substance use was described as both alleviating and causing distress and was often involved in intermittent homelessness, wherein people left their homes to access alcohol or khat and then were lost from their homes while intoxicated. Substance use problems are heavily stigmatized in this setting, both by the community and by those who have these problems themselves [48] and may, in conjunction with SMI, overwhelm the capacity of families to cope.

Coercive treatment of people with SMI was common and led to homelessness. Coercion was expressed in multiple forms, including physical restraint and in emotional pressure from the family to continue to take medication. Surveillance was another form of social coercion, and networks of surveillance extending to neighbors and other community members were perceived ambiguously by people with SMI, and reported to both cause and prevent homelessness. Previous studies from this area have indicated that there is little involvement of people with SMI in decision-making about their care [49] and human rights abuses in the community and at holy water treatment may drive people to run away from these settings.

Staying at holy water sites may be a form of ‘quasi-institutionalisation’ in this context, where people with SMI who cannot be managed in the home seek spiritual care and their caregivers get temporary reprieve as holy water attendants take on the burden of custodial care duties [50]. That people with SMI escaped from both holy water and psychiatric facilities indicates that the care administered in such settings does not adequately uphold the person’s sense of liberty. In the absence of mental health legislation, there is limited protection of an individual’s desire for autonomy and protection from human rights abuses [51].

The use of restraints was controversial, but both people with SMI and caregivers saw it as an alternative to homelessness, injury and death [52]. There is evidence that expanding integrated care within primary health care in the district helped to lower both the use of restraint and the rates of comorbid alcohol use disorder in this population over a one-year follow-up period [33]. Culturally-sensitive psychoeducation for people with SMI and their families should include modules related to both restraint and problematic substance use [53] and how to manage these issues in a way which upholds human rights.

### Pathways out of homelessness

While family conflict was a precipitant for homelessness, family and community intervention and support were crucial for people to return home. Families employed a flexible and wide-ranging array of measures to help family members with SMI who were homeless. The strategies used by families were adaptive and responsive to changes in the homeless person’s situation as well as factors including the ability to draw on available financial and other resources. While caregivers were motivated by a desire to help, their actions could still be coercive, particularly in regards to the use of restraint [52].

Those with long-term homelessness required more intensive strategies to return them to housing, largely because they were more detached from their families and communities of origin. Permanent housing was secured for several of the participants. Housing is a cornerstone of interventions for people with SMI who are homeless in HICs and the provision of immediate, long-term housing is considered to be an evidence-based best practice [22, 54]. In HICs, this housing is usually provided by the state with or without support from non-profit organizations [22]. In our study, we found that housing was procured not through government housing or another formal housing program, but instead through the work of concerned community members who mobilized and coordinated resources from a variety of sources.

Accessing health care was a stepping stone to leaving homelessness when it could be coupled with family and community support. Once family or community members witnessed a person with SMI improving with treatment, they appeared to be more willing to support them; people with SMI were also more motivated to reunite with their families and return home after receiving treatment. Community mobilization and support activities ranged from providing housing, to the facilitation of medical care, assistance with basic needs, and offering temporary shelter, including within Orthodox church compounds.

A number of people with intermittent homelessness simply returned home unaided. “Self-return” was related to the idea that homelessness could, at times, be an extension of an individual’s personal autonomy; indeed, some study participants expressed a preference for homeless life in comparison with the “prison”-like conditions experienced in their family homes, and others saw homelessness as a “way of life” and part of their identity.

### Meeting complex needs

Task-shared mental health care may not be sufficient to meet the complex needs of people who require extra support, particularly those who are the most ill and need more intensive resources [55]. There is potential for non-governmental organisations to step into this space to help such people [3, 56], as their work can be more intersectoral, community- and social-care based. Disability grants could alleviate some of the financial burden disproportionately experienced by families with SMI family members [28, 57].

Community based rehabilitation (CBR) [58] interventions have also been promoted as a way of providing more intensive, holistic support to people with SMI in the community [59]. However, there are currently no guidelines in CBR interventions for how to deal with homelessness. These could usefully include assessing and meeting urgent needs, followed by individualized joint care planning with the person with SMI when the situation stabilizes, and they are better able to participate. Having a home one finds acceptable is an essential condition for continuity of care [60]. Where possible, health extension workers or CBR workers could coordinate care across multiple sectors, including with government (‘*kebele’*) officials.

### Strengths and limitations

Strengths of the study include that we set our study in a context where a measure of trust and goodwill had been established between health workers and community members. This facilitated access to people with SMI and their caregivers and meant we were more likely to be welcomed than had our study been conducted independently of service provision. However, some people’s experiences of problems accessing care also affected their engagement with the interviews. Interviewing people with lived experience of both SMI and homelessness was a major strength of the paper and is the first time their voices have been featured so prominently in a publication from a LMIC setting (to the best of our knowledge). This is also the first study of people with SMI who have experienced homelessness in the context of the implementation of accessible integrated mental health care in a LMIC setting, highlighting its role in helping several community members to get out of homelessness. CS and KK were both outsiders to the community under study which may have impacted on the content of the interviews. Due to difficulties with accessing remote, rural areas, there was over-representation of participants who were based near the main town. Nonetheless, nesting the study within PRIME allowed us to identify people with SMI who had been homeless in the community. The availability of mental health care (delivered through the task-shared model) provides important insights on homelessness in the context of locally available mental health care but means that our findings may not be transferable to other settings.

Other issues impacting on the transferability of this study is that is was exploratory in nature and featured a relatively small number of participants. Another limitation is that we focused on pathways into and out of homelessness among people with SMI; it is likely that there are other determinants of pathways that could apply to all people and which are not listed here and could also apply to people with SMI (for example, larger migration trends or other macro-economic forces which cause uprooting from communities of origin).

### Implications for policy and practice

Overall, pathways into and out of homelessness in our setting were mediated more by individual, family, and community-level factors than by the structural factors which are often emphasized in HICs (for example, access to state-subsidised medical and social care and housing). This may be due in part to the fewer structural-level assets available in this setting, as well as the stronger cultural influence of family and community.

Ending long-term homelessness in this setting will likely require transitional housing in addition to medical and social support, with family reunification and financial support as a second step [61]. There is precedence for such a stepped approach in urban India with the work of the Banyan NGO [62], where women with SMI are first given medical and rehabilitative care before overtures are made to reconnect women with their families of origin. A recent study from Mozambique [63] demonstrated the proof of concept for the potential for psychiatric institutions to act as intermediate housing and rehabilitation for people with SMI in a LMIC context, however the study only had a follow-up period of 3 months and there are few psychiatric beds per capita in LMIC settings, so this is not feasible to address homelessness on the societal-level.

Intermittent homelessness might be better addressed by focusing on building up the family unit and psychoeducation [64]. Modified, culturally appropriate “critical time interventions” [65], wherein intensive, time-limited support is provided during the transition period back into domiciled life may be appropriate for both types of homelessness.

Ultimately, this study shows the profound contribution of the community in assisting people with SMI to get out of homelessness and this is an important finding for others planning mental health care services in low resource settings. Different types of service responses will need to address the different types of homelessness and must take into account personal needs and preferences and be sensitive to uphold the person’s sense of liberty and participation in care planning. Future work must focus on identifying and then supporting and bolstering strategies already used by those with SMI, their families and communities.

## Supplementary Information

**Additional file 1 Table S1.** Living situations for study participants at the time of interview.

## Data Availability

The datasets generated during and/or analyzed during the current study are not publicly available due to ethical considerations and for protection of participant privacy but are available from CS upon reasonable request.

## Abbreviations

IDI: In-depth interview
LMIC: Low and middle income countries
PRIME: Programme to Improve Mental Healthcare
SMI: Severe mental Illness
SNNPR: Southern Nations, Nationalities and Peoples’ Region
