# Supplementary material for: Pathways into and out of homelessness among people with severe mental illness in rural Ethiopia: a qualitative study
Source: BMC Public Health. 2021 Mar 22;21:568. doi: 10.1186/s12889-021-10629-8 (PMC7986271; doi:10.1186/s12889-021-10629-8)
Supplement: Supplementary file 1 — Additional file 1 Table S1. Living situations for study participants at the time of interview. [file 12889_2021_10629_MOESM1_ESM.docx]

Supplementary table 1: Living situations for study participants at the time of interview

| Interview | Gender | Living situation at time of interview |
| --- | --- | --- |
| IDI 3 | Female | Living in a house procured for her by PRIME with her son |
| IDI 7 | Male | Living in his caregiver’s house |
| IDI 11 | Male | Living with his family |
| IDI 12 | Male | Wandering (homeless) at the time of interview |
| IDI 13 | Male | Living with his family |
| IDI 14 | Male | Living with his family |
| IDI 16 | Male | Wanders in the street and drinks alcohol |
| IDI 18 | Male | Living with his family |
| IDI 19 | Male | Living with his family |
| IDI 20 | Male | Living with his family, bed-ridden at the time of interview |
| IDI 22 | Male | Living with his family |
| IDI 23 | Male | Living with his family |
| IDI 24 | Male | Living with his family |
| IDI 25 | Female | Living in a rented house |
| IDI 26 | Female | Living with her family |
